# Quantification of Microvascular Density of the Optic Nerve Head in Diabetic Retinopathy Using Optical Coherence Tomographic Angiography

**DOI:** 10.1155/2020/5014035

**Published:** 2020-04-29

**Authors:** Jianfeng Huang, Bodi Zheng, Yingyi Lu, Xiaoya Gu, Hong Dai, Tong Chen

**Affiliations:** Department of Ophthalmology, Beijing Hospital, National Center of Gerontology; Institute of Geriatric Medicine, Chinese Academy of Medical Sciences, No. 1 Dahua Road, Dongcheng District, Beijing 100730, China

## Abstract

**Aims:**

To quantify the capillary density of the optic nerve head in healthy control eyes and different stages of diabetic retinopathy (DR) eyes and identify the parameters to detect eyes with or without DR using optical coherence tomographic angiography (OCTA).

**Methods:**

In this cross-sectional study, 211 eyes of 121 participants with type 2 diabetes with different stages of DR or without DR and 73 eyes of 38 healthy age-matched controls were imaged by OCTA. Radial peripapillary capillary (RPC) plexus density and retinal nerve fiber layer (RNFL) thickness were examined. The mixed model binary logistic regression model was used to identify the parameters to detect eyes with or without DR. The area under the receiver operating characteristic (ROC) curve was calculated.

**Results:**

RPC density decreased significantly in diabetic patients without DR compared with the healthy controls, and it was negatively correlated with the severity of DR (*P* < 0.01). RPC density was a significant parameter to distinguish diabetic eyes with or without DR (*P* < 0.01). The area under the ROC curve was 0.743.

**Conclusions:**

Quantification of RPC density by OCTA provides evidence of microvascular changes in the optic nerve in diabetic patients. RPC density can serve as a possible biomarker in detecting eyes with DR. Larger cohort studies need to support this statement.

## 1. Introduction

Optical coherence topographic angiography (OCTA) is now emerging as a noninvasive method to detect the blood flow and microvasculature of retina without intravenous dye injection [1–3]. OCTA has been used to discriminate between healthy eyes and eyes of diabetic patients with no diabetic retinopathy (DR) [4–8]. Some authors found that the macular parameters of OCTA are associated with the severity of DR [9], suggesting that OCTA can serve as an early detection tool of DR [10].

Based on previous studies, we know that DR does have not only microvascular changes but also neurodegeneration [11, 12]. The mostly used parameter to evaluate the neurodegeneration on OCT is retinal nerve fiber layer (RNFL) thickness, and now with the help of OCTA, we can also evaluate the microvascular impairment around the optic disc by measuring radial peripapillary capillary (RPC) density [13, 14]. RPC density on OCTA can be automated divided into two different areas, the peripapillary area and the inside optic disc area. Recent studies showed that RPC density of DR patients was significantly reduced compared with that of healthy controls. The vessel density of RPC is correlated with the severity of DR [15]. However, the number of studies on quantification of the vessel density and the neurodegeneration in and around the optic disc using OCTA is still limited.

The aim of our study was to evaluate the vessel density in the eyes of patients with type 2 diabetes, find effective parameters to distinguish eyes with or without DR changes, and detect preclinical DR.

## 2. Materials and Methods

This was a cross-sectional observational study. Patients with type 2 diabetes were recruited in the Department of Ophthalmology at Beijing Hospital, Beijing, from July 16, 2018, to June 18, 2019. The tenets of the Declaration of Helsinki [16] were followed, and approval was obtained from the research ethics committee of Beijing Hospital. Written informed consent to participate in the study was obtained from all individuals after explaining all procedures.

Inclusion criteria were eyes of patients with T2DM without DR, eyes with different stages of nonproliferative diabetic retinopathy (NPDR). DR stages were confirmed based on the international classification system [17]. The grading of no DR, mild-to-moderate NPDR, and severe NPDR was provided by a single retinal specialist by clinical examination. The information about previous medical history and ocular conditions was obtained by interviewing the participants. Exclusion criteria were eyes with any history of ocular injury, ocular surgery, and other retinal and optic nerve diseases that may confound the results. Participants with opaque optic media and high refractive error (more than 6 diopters of sphere or more than 3 diopters of cylinder) were also excluded. Only individuals with a scan quality above 6/10 were eligible.

Information on age, gender, diabetic duration, and hemoglobin A1c (HbA1c) level were collected based on the patient medical records. The LogMAR best-corrected visual acuity (BCVA) was noted for each eye.

**Figure 1:**
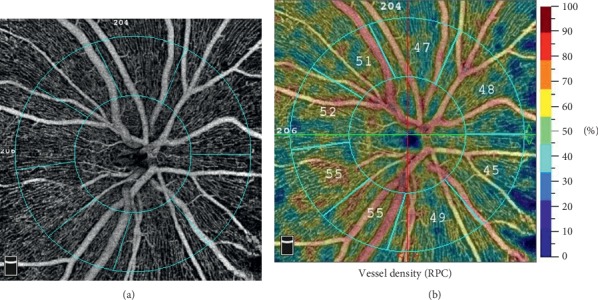
Radial peripapillary capillary (RPC) density measurement on OCTA. (a) Angiographic image of the 4.5 *∗* 4.5 rectangle scan of the ONH. The inside optic disc area is the area surrounded by the inner blue circle; the peripapillary region area is between the two blue rings; (b) vessel density showed by the colour maps. The different numbers of the outer ring represent the capillary density of the peripapillary area.

OCTA images were obtained using the RTVue XR Avanti device with AngioVue 2.0 (Optovue Inc., Fremont, CA, USA). A 4.5 *∗* 4.5 mm scan centered on the optic nerve head was performed. The built-in Angio Analytics software (version 2017.1.0.155; Optovue, Inc.) was used to evaluate vessel density and RNFL thickness. The software defines the peripapillary region as a 1.0 mm wide round annulus extending from the optic disc boundary and the inside optic disc area as a 2.0 mm diameter circle surrounding the optic disc. The peripapillary and the inside optic disc areas together composite the 4.0 mm-diameter round whole image. The RPC segment extends from the inner limiting membrane to the nerve fiber layer. The vessel density is defined as the percentage of the area occupied by the vessels in the corresponding region (Figure 1). The software calculated the vessel density. Simultaneously, the average RNFL thickness for the peripapillary area was recorded.

For statistical analysis, IBM SPSS statistics version 21 (IBM SPSS Statistics; IBM Corporation, Chicago, IL, USA) was used. A one-way ANOVA was applied to compare the continuous variables among the four groups (healthy controls, NDR, mild-to-moderate NPDR, and severe NPDR), and LSD or Dunnett T3 post hoc analysis was performed to evaluate the significant differences. Categorical variables were analyzed using a chi-squared test. Spearman and Pearson tests were used to examine nonlinear and linear correlations between the OCTA parameters and different DR groups (healthy controls, NDR, mild-to-moderate NPDR, and severe NPDR) and ages. A partial correlation test was performed to analyze the correlations between the OCTA parameters and DM duration, HbA1c and BCVA by adjusting different DR severities. A mixed model binary logistic regression model was employed to find out the related parameters that can distinguish the eyes with or without diabetic retinal changes in the diabetic patients. In this model, we included random effects for the correlation of two eyes from the same participants on the OCTA outcome measures, as most participants had bilateral imaging. For determining the diagnostic value, the ROC curve was then generated based on the binary logistic regression model. The area under the curve (AUC), sensitivity, and specificity were reported. A *P* value <0.05 was considered statistically significant.

## 3. Results

73 eyes of 38 healthy participants and 211 eyes of 121 DM patients were imaged. The detailed characteristics of the participants are shown in Table 1.

Table 2 shows parameters of RPC densities and RNFL thicknesses in different groups. The RPC densities of the whole image, inside optic disc area, and peripapillary area were significantly different among the four groups. No significant difference of RNFL thickness was found.

The post hoc analysis in Supplementary Table S1 demonstrates that RPC density of all DR groups was significantly different from that of the healthy controls only in the peripapillary area, and severe NPDR had significantly reduced vessel density in all the considered scans with regard to lower categories of DR and healthy controls.

Correlation coefficients for different DR severities and association of variables with RPC density in OCTA are shown in Table 3.

**Figure 2:**
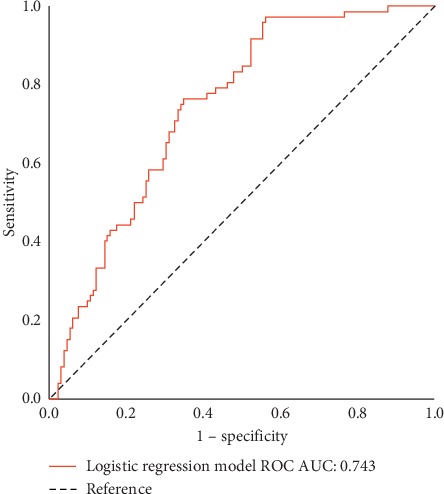
Receiver operating characteristic curve (ROC) for the mixed model binary logistic regression of radial peripapillary capillary (RPC) density in the peripapillary area between eyes with or without diabetic retinopathy (DR). AUC indicates area under the curve.

Significant correlations between DR severity and RPC densities in all imaged areas were observed (Table 2). A negative weak correlation between age, HbA1c, DM duration, and the OCTA parameters was found except for DM duration with inside optic disc area RPC density. No significant correlation was found between BCVA and the RPC densities. RPC density in the peripapillary area was found to be an independent predictor (odds ratio, 0.78; 95% CI: 0.713–0.870; *P* < 0.01). The AUC of the ROC curve was 0.743 (95% CI: 0.676–0.810; *P* < 0.01) (Figure 2).

## 4. Discussion

In this study, we evaluated the use of OCTA for the quantitative measurement of microvascular changes of the optic nerve head in healthy participants and patients with DM without DR and with mild-to-severe NPDR. We found that RPC density, a quantitative parameter of microvascular change of ONH, was significantly reduced in patients with DM regardless of DR compared with the healthy participants. We also noticed that RPC density decreased with the increasing grading of NPDR. Our finding was inconsistent with the finding of Rodrigues et al. [18], who stated that no graded effect was observed in RPC density according to DR severity. Nevertheless, our study and other previous studies [13–15, 19, 20] suggested that microvascular changes of RPC density was affected by diabetes.

In order to find out if RPC density can serve for early detection of preclinical stage of DR, we compared RPC density between healthy controls and diabetic patients with no DR signs. RPC density was significantly decreased in diabetic patients with no DR compared with the healthy controls, which fit well with the recent publications [14, 20]. Besides, our results indicated that RPC density could also be used for detection of the different degrees of DR changes in diabetic patients.

We found that HbA1c and DM duration were correlated with RPC density. This result was not in agreement with the previous studies [18, 20], in which HbA1c and DM duration were not found to be associated with RPC density. The reason for the difference may lie on the different groups of patients we enrolled. In contrast with the previous studies, severe NPDR patients included in our study might contribute to the different findings. Though Liu et al. [15] enrolled severe NPDR patients in their study, they did not analyze the correlations between Hb1Ac, DM duration, and RPC density, respectively.

In our study, no significant difference of RNFL thickness was found among the different DR severity groups. Previous studies [14, 15] had similar results to ours, but some authors found significant RNFL thickness thinning in eyes with DR compared with the healthy controls [21, 22]. The lack of significant difference in RNFL thickness in the peripapillary region among the groups observed herein could possibly be attributed to the structural changes in the retinal tissue in the peripapillary region caused by intracellular and extracellular edema, exudation, or glial fibrillary degeneration around the optic disc.

As implied by the previous studies, DR is a neurovascular disease [23, 24]. Alterations in the capillary density around the optic nerve head may be the start point of the neurodegeneration process of DR. Possible relationship between the RPC network and the macular vascular network has been indicated [25]. The reduction of capillary density around the optic nerve head may be a reflection of ischemia in DR. Quantification of RPC density may contribute to a better understanding of the neurodegenerative pathogenesis of DR.

The limitations of our study include the cross-sectional design, the relatively small sample, and the automatic segmentation applied in density calculations which may lead to segmentation errors. This study did not analyze the effect of hypertension and other factors that may account for the change of RPC density. However, the strength of this study include that it contained all stages of NPDR and analyzed each part of the imaging area of RPC density.

## 5. Conclusions

RPC density is negatively correlated with DR severity. Reduction of capillary density in the peripapillary area can be introduced as a biomarker in early detection of DR though further and larger cohort studies should be needed to support the statement. OCTA findings of the capillary density of the ONH provide valuable insights of the neurovascular changes of DR.

## Figures and Tables

**Table 1:** Demographic characteristics of patients and healthy controls.

Characteristics	Group				*P* value
	Healthy controls	No DR	Mild-to-moderate NPDR	Severe NPDR	
Patients, no.	38	45	36	40	

Eyes, no.	73	80	68	63	

Age, y					0.246
Mean ± SD	55.0 ± 16.0	58 ± 12.0	57.9 ± 10.8	60.7 ± 8.5	

Sex					0.434
Female, no. (%)	19 (50%)	15 (25%)	13 (36.1%)	17(42.5%)	
Male, no. (%)	19 (50%)	30 (75%)	23 (63.9%)	23(57.5%)	

DM duration, *y*					0.207
Mean ± SD	N/A	12.2 ± 7.3	13.0 ± 7.1	15.0 ± 6.9	

HbA1c, %					0.704
Mean ± SD	N/A	8.7 ± 1.7	8.9 ± 1.4	9.1 ± 2.1	

LogMAR BCVA					<0.01
Mean ± SD	0.04 ± 0.07	0.12 ± 0.2	0.29 ± 0.30	0.54 ± 0.37	
Anti-VEGF					
Treatment	N/A	0 (0%)	3 (4.4%)	7 (11.1%)	

DR, diabetic retinopathy; NPDR, nonproliferative diabetic retinopathy; DM, diabetes mellitus; HbA1c, hemoglobin A1c; BCVA, best-corrected vision acuity; VEGF, vascular endothelium growth factor.

**Table 2:** One-way ANOVA for RPC density and RNFL thickness in OCTA.

RPC density	Group				F value	*P* value
	Healthy controls	No DR	Mild-to-moderate NPDR	Severe NPDR		
Whole image, %						
Mean ± SD	49.9 ± 2.3	49.0 ± 2.8	47.3 ± 3.9	45.5 ± 3.4	26.2	<0.01
Inside optic disc, %						
Mean ± SD	50.8 ± 5.8	49.0 ± 4.9	49.5 ± 6.7	45.8 ± 5.9	8.8	<0.01
Peripapillary,%						
Mean ± SD	53.1 ± 2.6	51.7 ± 3.4	49.8 ± 4.6	47.4 ± 4.2	29.6	<0.01
RNFL thickness, *μ*m						
Mean ± SD	115.4 ± 10.5	115.1 ± 23.3	109.4 ± 20.0	112.7 ± 20.3	1.5	0.22

RPC, radial peripapillary capillary; RNFL, retinal nerve fiber layer; OCTA, optic coherence tomographic angiography; DR, diabetic retinopathy; NPDR, nonproliferative diabetic retinopathy.

**Table 3:** Correlation coefficients for DR severity and association of variables with RPC density in OCTA.

	RPC density		
	Whole image	Inside optic disc	Peripapillary
DR severity			
*R* value^*a*^	−0.536	−0.269	−0.541
*P* value	<0.01	<0.01	<0.01

Age			
*R* value^*b*^	−0.132	−0.237	−0.142
*P* value	0.027	<0.01	0.017

DM duration			
*R* value^*c*^	−0.191	−0.024	−0.209
*P* value	<0.01	0.734	<0.01

HbA1c			
*R* value^*c*^	−0.188	−0.141	−0.146
*P* value	0.01	0.045	0.037

BCVA			
*R* value^*c*^	−0.053	−0.031	−0.042
*P* value	0.447	0.662	0.547

*a*, Spearman coefficient *R* value; *b*, Pearson coefficient *R* value; *c*, partial correlation coefficient *R* value; RPC, radial peripapillary capillary; DR, diabetic retinopathy; DM, diabetes mellitus; HbA1c, hemoglobin A1c; BCVA, best-corrected vision acuity.

## Data Availability

The SPSS statistics data document data used to support the findings of this study are included within the supplementary information file.

## Supplementary Materials

Supplementary Materials Table S1: post hoc analysis of the ONH RPC density in different groups
