# Supplementary material for: Quantification of Microvascular Density of the Optic Nerve Head in Diabetic Retinopathy Using Optical Coherence Tomographic Angiography
Source: J Ophthalmol. 2020 Apr 29;2020:5014035. doi: 10.1155/2020/5014035 (PMC7206883; doi:10.1155/2020/5014035)
Supplement: Supplementary Materials — Table S1: post hoc analysis of the ONH RPC density in different groups [file 5014035.f1.pdf]

Supplementary Table

Table S1 Post-hoc analysis of the ONH RPC density in different groups

RPC density	Homogeneity Test, p value	Post-hoc method	Group	Group(compared)	P value
Whole image	0.002	Dunnett T3	Control	No DR	0.142
				Mild-Moderate NPDR	<0.01
				Severe NPDR	<0.01
			No DR	Control	0.142
				Mild-Moderate NPDR	0.022
				Severe NPDR	<0.01
			Mild-Moderate NPDR	Control	<0.01
				No DR	0.022
				Severe NPDR	0.035

			Severe NPDR	Control	<0.01
				No DR	<0.01
				Mild-Moderate NPDR	0.035
Inside optic disc	0.101	LSD	Control	No DR	0.062
				Mild-Moderate NPDR	0.174
				Severe NPDR	<0.01
			No DR	Control	0.062
				Mild-Moderate NPDR	0.655
				Severe NPDR	0.001
			Mild-Moderate NPDR	Control	0.174
				No DR	0.655

				Severe NPDR	<0.01
			Severe NPDR	Control	<0.01
				No DR	<0.01
				Mild-Moderate NPDR	<0.01
Peripapillary	0.002	Dunnett T3	Control	No DR	0.041
				Mild-Moderate NPDR	<0.01
				Severe NPDR	<0.01
			No DR	Control	0.041
				Mild-Moderate NPDR	0.029
				Severe NPDR	<0.01
			Mild-Moderate NPDR	Control	<0.01
				No DR	0.029

				Severe NPDR	0.012
			Severe NPDR	Control	<0.01
				No DR	<0.01
				Mild-Moderate NPDR	0.012
